# Complete PSA Remission without Adjuvant Therapy after Secondary Lymph Node Surgery in Selected Patients with Biochemical Relapse after Radical Prostatectomy and Pelvic Lymph Node Dissection

**DOI:** 10.1155/2012/609612

**Published:** 2011-06-26

**Authors:** Alexander Winter, Jens Uphoff, Rolf-Peter Henke, Friedhelm Wawroschek

**Affiliations:** ^1^Department of Urology and Paediatric Urology, Hospital Oldenburg, 26133 Oldenburg, Germany; ^2^Institute of Pathology Oldenburg, 26122 Oldenburg, Germany

## Abstract

*Introduction*. To evaluate whether secondary resection of lymph node (LN) metastases (LNMs) can result in PSA remission, we analysed the PSA outcome after resection of LNM detected on PET/CT in patients with biochemical failure. *Materials and Methods*. 11 patients with PSA relapse (mean 3.02 ng/mL, range 0.5–9.55 ng/mL) after radical prostatectomy without adjuvant therapy were included. Suspicious LN (1–3) detected on choline PET/CT and nearby LN were openly dissected (09/04–02/11). The PSA development was examined. Histological and PET/CT findings were compared. *Results*. 9 of 10 patients with histologically confirmed LNM showed a PSA response. 4 of 9 patients with single LNM had a complete permanent PSA remission (mean followup 31.8, range 1–48 months). Of metastasis-suspicious LNs (14) 12 could be histologically confirmed. The additionally removed 25 LNs were all correctly negative. *Conclusions*. The complete PSA remissions after secondary resection of single LNM argue for a feasible therapeutic benefit without adjuvant therapy. For this purpose the choline PET/CT is in spite of its limitations currently the most reliable routinely available diagnostic tool.

## 1. Introduction

In prostate cancer the long relapse-free survival of patients with 1-2 LNM even without adjuvant therapy in the primary situation [1, 2] argues for a feasible therapeutic benefit by resection of LNM especially in case of minimal lymphatic dissemination. To evaluate whether the sole secondary resection of LNM can result in a prostate-specific antigen (PSA) remission, we analysed the PSA outcome after targeted resection of LNM detected via choline positron emission tomography (PET)/computed tomography (CT) in patients with biochemical failure after radical retropubic prostatectomy.

In our first studies of PET/CT-guided secondary LN surgery, we reported on the outcome of all in all 8 patients with LNM detected by using [^11^C]choline PET/CT without adjuvant therapy [3, 4]. 3 of 6 patients with single LN recurrence showed a complete PSA remission without adjuvant therapy up to 32 months. Now we wanted to update the results of secondary LN dissection in consideration of more patients and a longer followup. Moreover, in our present study patients with LNM detected by using [^18^F]fluoroethylcholine were included too.

Former studies of others could give no evidence for PSA-remission after the sole secondary resection of LNM, whereas a resection of LNM was followed by adjuvant therapy [5, 6] and patients without adjuvant therapy were monitored for only a short time, respectively [7].

The integrated [^11^C]choline and [^18^F]fluoroethylcholine PET/CT provides the opportunity to detect small LNM (>5 mm) in prostate cancer with exact topographic allocation and so the targeted resection of LNM. In contrast, the computed tomography (CT) and the conventional magnetic resonance imaging (MRI) are not applicable for early detection of LN recurrence. The lymphotropic nanoparticle-enhanced MRI can detect smaller LNM (>2 mm) [8] but has not been approved for routine diagnostics. 

## 2. Materials and Methods

### 2.1. Patients

11 consecutive patients (mean age 62 years, range 49–78 years) with 1–3 LNM detected by using [^11^C]choline PET/CT (*n* = 9) or [^18^F]fluoroethylcholine PET/CT (*n* = 2) in case of PSA failure (mean 3.02 ng/mL, range 0.5–9.55 ng/mL) were included. All had a PSA increase or persistence after operative therapy which was performed between 3 months and 9 years ago. In 10 patients a radical retropubic prostatectomy with pelvic LN dissection (PLND) and in one patient only a radical retropubic prostatectomy were carried out. One patient had received a sentinel guided PLND (sPLND) on both sides of the pelvic and also an extended PLND (ePLND) on the right side because of an advanced tumor and another one only sPLND in our clinic. The remaining patients had received conventional PLND, carried out by other institutions. There had to be negative margins and no clue for a local relapse or distant metastasis. The patients were informed that there is no conclusive data concerning survival benefit after secondary LN surgery in written and oral form, and signed an informed consent. 

### 2.2. Choline PET/CT Imaging

**Figure 1:**
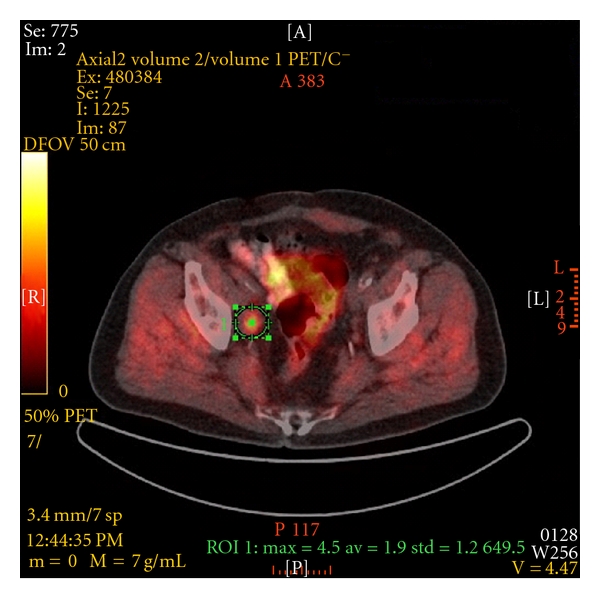
Integrated [^11^C]choline PET/CT shows a single LNM in the right iliac region. The LNM was confirmed histopathologically after secondary resection. (Source: Clinic of Nuclear Medicine and Institute of Clinical Radiology, University Hospital Muenster, Germany).

All [^11^C]choline or [^18^F]fluoroethylcholine PET/CT studies were performed with integrated PET/CT systems externally in four centres with a high level of expertise. Experienced radiologists and nuclear medicine specialists evaluated the images to anatomically localize the sites of pathologic choline uptake. The diagnosis of tumor positive LN on PET/CT images was based on the visual evidence of the presence of focal increased choline uptake on PET images, whose location corresponded to LN on CT images (Figure 1). 

### 2.3. Surgical Procedure, PSA Development, and Histological Evaluation

The LN/LNM detected by use of choline PET/CT and the nearby LN were openly dissected by two high-volume surgeons (09/2004–02/2011). The PSA development was monitored up to 48 months (mean 18.6, range 1–48 months) postoperatively. The primary histological diagnosis was made on hematoxylin and eosin-stained sections. Immunohistochemical staining of cytokeratins was performed to verify micrometastases. In one case, additional antibodies against PSA, prostate specific acid phosphatase, p504s, and the proliferation marker Ki67 were employed for typing of the metastatic tissue. The histological findings were compared with the PET/CT results. 

## 3. Results

A summary of the patient characteristics is shown in Table 1. The mean PSA value at the date of the choline-PET/CT examination was 3.02 ng/mL (range 0.5–9.55 ng/mL).

In 10 of 11 patients the metastasis-suspicious LN detected by means of PET/CT could be completely removed. They were also histologically positive. In one patient with two metastasis-suspicious LN detected on PET/CT, only one histological negative LN could be resected because of severe cicatrization. A further 25 (mean 2.3, range 0–10) adjacent PET/CT negative LNs were dissected and negative for cancer. In one case, neighbouring LN could not be removed, because only three months ago this patient received a sPLND and ePLND on the concerning side in our clinic. In another patient only cicatricial tissue could be removed in addition to one LNM after radiotherapy. In the same patient a small lesion of the ureter necessitated a secondary ureteral stenting. In all other cases the intra- and postoperative courses were without complications.

**Figure 2:**
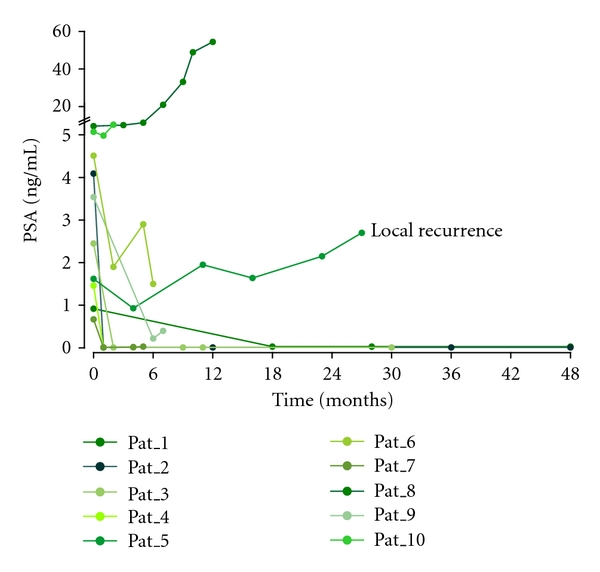
PSA development after secondary resection of LNM without adjuvant therapy.

After the secondary LN resection, 9 of 10 patients with histologically confirmed LNM showed a PSA response. 4 of 9 patients with single metastases had a lasting complete PSA remission (<0.01 ng/mL (*n* = 3), <0.03 ng/mL (*n* = 1)) without adjuvant therapy. The maximum followup of these patients was 48 months (mean 31.8 months, range 1–48 months). The other 5 patients with single LNM initially showed a PSA remission, 4 of them a partial incomplete remission. In one of these cases, a local recurrence was detected in the course of the study by means of PET/CT and MRI. In two other patients with incomplete remission already a tumorous infiltration of the adjacent tissue was histologically detected. In the patient without PSA response, 3 LNMs were histologically confirmed. The PSA development of all patients can be seen in Table 2 and Figure 2. 

## 4. Discussion

The studies dealing with the secondary resection of LNM existing so far could give no evidence concerning the PSA response. The patients were either treated postoperatively with hormones or radiation [5, 6] or were monitored without adjuvant therapy for a maximum of four months, respectively [7]. In our present study 4 patients with single LNM even showed a complete and lasting PSA remission, over a maximum followup of 48 months, without an adjuvant treatment. These results confirm our previous data of the complete PSA remission in patients with single LN recurrence after secondary LN dissection [4].

Whether patients can benefit therapeutically by the removal of LNM in prostate cancer is still inconclusive. In our group the complete PSA remissions after secondary resection of single LNM argue for a feasible therapeutic benefit. Nevertheless, the small number of cases and the comparative short followup is a limitation of our study. The long-term outcome of patients undergoing PET/CT guided secondary resection of LNM remains to be seen. However, observations in the primary situation support a therapeutic benefit especially for patients with minimal lymphatic dissemination. Several reports suggest that ePLND increases the likelihood of finding positive nodes and improves biochemical relapse free survival [1, 9]. In the study of Daneshmand et al. [1] LN-positive patients had a progression-free survival of 70% (one positive LN), respectively, 73% (two positive LNs) after ten years. von Bodman et al. showed [2] that the time (median) to relapse without adjuvant therapy was 59 months (1 LNM), 13 months (2 LNMs), and 3 months for patients with 3 LNMs. After 24 months 79% (one positive LN, Gleason-Score ≤7) or 29% (≥two positive LN, Gleason-Score ≥8) were free of biochemical relapse. Catalona et al. [10] reported that LN-positive patients without an adjuvant treatment developed no biochemical recurrence in 75% over six years and up to 58% over seven years. A population-based case-cohort study indicates a possible therapeutic benefit of PLND in node negative patients [11]. However, several studies demonstrated that in histologically negative LN tumor cells could be detected by real-time reverse transcriptase PCR [12, 13].

A limitation of secondary LN surgery in PCa is the limited sensitivity of the currently available imaging, especially in detection of small LNM. Contrary to conventional MRI and CT, the PET ([^11^C]choline, [^11^F]choline) offers key benefits in detecting LNM in the primary and recurrent diagnosis of PCa foci of sizes up to 5 mm [14, 15]. Nonetheless, the value of this method is limited because of the frequency of smaller metastases (<5 mm) [16, 17]. Other authors have also shown the method's inaccuracy in detecting lesions smaller than 1 cm [18–20], but CT and MRI are far more unreliable in these cases [21]. The lymphotropic nanoparticle-enhanced MRI can detect smaller LNM (>2 mm) [8] but has not been approved for routine diagnostics. In the future, the diffusion-weighted MRI could provide additional information on tumor pathophysiology compared to the standardized uptake values (SUV) in choline PET/CT. In a pilot study of Beer et al., [22] the apparent diffusion coefficient value in diffusion-weighted MRI and the SUV in PET showed a highly significant inverse correlation in LN diagnostics.

Whether choline PET/CT offers the basis of early treatment decisions in patients with PSA failure after radical prostatectomy is a subject of ongoing discussion. Picchio et al. suggest that the routine use of choline PET/CT cannot be recommended for PSA values <1 ng/mL [23]. However, patients with local recurrence after radical prostatectomy are best treated by salvage radiotherapy when the PSA serum level is <0.5 ng/mL. We could detect positive findings with a very low PSA value (≥0.67 ng/mL). Also Scattoni et al. [5] and others [24, 25] have shown positive results in patients with very low PSA levels (<1 ng/mL). In a study of Castellucci et al. it was possible to detect recurrent disease in 28% of patients with PSA <1.5 ng/mL by PET/CT [26]. In 21% of the patients distant unexpected metastases were detected by PET/CT. In those cases an unnecessary local radiotherapy can be avoided. 

Our study shows a complete correlation between [^11^C]choline PET/CT as well as [^18^F]fluoroethylcholine PET/CT and histological findings in patients with single LNM (specificity 100%) and a specificity of 86% over all patients. With respect to the method, conclusions on sensitivity of choline PET/CT cannot be given. 

## 5. Conclusions

Especially patients with minimal LNM seem to benefit from the secondary removal of LNM in prostate cancer. The here observed lasting, complete PSA remissions after secondary resection of single LNM appear to have a feasible therapeutic benefit without adjuvant therapy. For this purpose, the [^11^C]choline or [^18^F]fluoroethylcholine PET/CT is, despite its limitations, currently the most reliable routinely available diagnostic tool. Whether the secondary resection of LNM has an influence on the course of disease or could even be curative must be demonstrated in further studies in consideration of more patients and a long-term followup. 

## Figures and Tables

**Table 1:** Summary of the patient characteristics.

Patient	Age (yr)	Primary treatment	Initial tumour stage	Gleason score	Hormonal therapy after primary treatment	Radiotherapy after primary treatment	PSA initial (ng/mL)	PSA1 (ng/mL)	PET/CT positive LN
1	61	RPE + PLND	pT3a pN0 cM0 R0	3 + 4	−	−	4.13	0.92	1
2	59	RPE + PLND	pT2c pN0 cM0 R0	4 + 3	+	−	26.7	4.09	1
3	64	RPE + Splnd + ePLND right	pT3a pN1 cM0 R0	4 + 3	−	−	16.0	2.45	1
4	68	RPE	pT3a pN0 cM0 R0	?	−	+	9.9	1.64	1
5	78	RPE + PLND	pT3b pN0 cM0 R0	3 + 4	−	−	3.2	1.62	1
6	59	RPE + PLND	pT3a pN0 cM0 R0	4 + 3	+	−	7.6	4.51	1
7	49	RPE + PLND	pT3b pN0 cM0 R0	4 + 5	−	−	4.0	0.67	1
8	61	RRP + PLND	pT3a pN0 cM0 R0	5 + 5	+	+	?	9.55	3
9	53	RRP + PLND	pT3a pN0 cM0 R1	4 + 4	−	+	36.0	3.54	1
10	75	RRP + sPLND	pT3a pN0 cM0 R1	4 + 3	−	+	5.94	3.77	1
11	55	RRP + PLND	pT3b pN1 cM0 R0	4 + 3	−	−	5.08	0.5	2

mean	62							3.02	

PSA initial: PSA at primary diagnosis; PSA1: PSA at time of PET/CT diagnosis.

**Table 2:** PSA development after secondary resection of LNM without adjuvant therapy.

Patient	PSA1 (ng/mL)	PSA2 (ng/mL)	Follow up (month)
1	0.92	<0.03	48
2	4.09	<0.01	48
3	2.45	<0.01	30
4	1.46	<0.01	1
5	1.62	2.7	27
6	4.51	1.5	6
7	0.67	0.03	5
8	9.55	54.46	12
9	3.54	0.4	7
10	3.77 (6.51*)	10.3	2
11	No LNM histologically confirmed		

PSA1: PSA at time of PET/CT diagnosis; PSA2: PSA after resection of LNM; *preoperative, 3 months after PET/CT.
